# Efficacy of Stem Cell Therapy for Interstitial Cystitis/Bladder Pain Syndrome: A Systematic Review and Meta‐Analysis of Preclinical Studies

**DOI:** 10.1155/sci/4556842

**Published:** 2026-10-25

**Authors:** Tao Yang, Wu Tang, Yuanzhi Li, Junchao Wu, Huitao Wang, Xiang Zhao, Kewei Fang

**Affiliations:** ^1^ Department of Urology The Second Affiliated Hospital of Kunming Medical University Kunming 650000 China kmmc.cn

**Keywords:** animal models, bladder pain syndrome, interstitial cystitis, mesenchymal stem cells, meta-analysis, preclinical evidence, stem cells

## Abstract

**Background:**

Interstitial cystitis/bladder pain syndrome (IC/BPS) is a refractory urological disorder for which effective disease‐modifying treatments remain limited. This meta‐analysis evaluates the preclinical efficacy of stem cell interventions for IC/BPS and establishes an evidence‐based roadmap for clinical translation.

**Methods:**

A systematic search of PubMed, Embase, Web of Science, and Cochrane Library was performed (up to July 8, 2025). Risk of bias was appraised using the SYRCLE framework. Our outcomes focused on bladder histopathology, local inflammatory markers, urodynamic profiles, voiding patterns, and nociceptive behaviors.

**Results:**

Eighteen studies (27 comparisons) evaluating interventions based on mesenchymal stem/stromal cells (MSCs) in rat models were included. Stem cell therapy significantly alleviated histopathological damage by reducing mast cell infiltration (SMD = −2.42), collagen deposition (SMD = −2.13), and apoptosis (SMD = −3.09), while suppressing proinflammatory cytokines (TNF‐α, IL‐6, IL‐1β). Functionally, interventions increased bladder capacity (SMD = 2.69), prolonged intercontraction intervals (SMD = 2.39), and markedly alleviated visceral pain (SMD = −3.19). Subgroup analyses identified human embryonic stem cell (hESC)‐derived multipotent mesenchymal stem cells (M‐MSCs) as the most extensively validated reference type, though other cell types exhibited phenotype‐specific potential. Furthermore, a dose of 1 × 10^6^ cells/rat, localized bladder wall injection (BWI), and allogeneic sources were identified as the most favorable preclinical parameters among the evaluated studies.

**Conclusions:**

Stem cell therapy—predominantly represented by MSCs—represents a promising candidate intervention with potential disease‐modifying properties for IC/BPS in preclinical models. To inform future trial designs, we suggest a preliminary, theoretically scaled human equivalent dose window of 2.0 × 10^7^ to 5.0 × 10^7^ cells, preferentially administered via localized BWI. By exploring optimal cellular parameters and translational barriers, this study provides an evidence‐based reference framework to guide the transition of stem cell therapy from bench to bedside.

## 1. Introduction

Interstitial cystitis/bladder pain syndrome (IC/BPS), characterized by persistent pelvic pain perceived to be related to the urinary bladder, accompanied by urinary frequency and urgency, remains a highly refractory urological disorder [1–3]. The underlying pathophysiology is multifactorial, involving urothelial barrier dysfunction, chronic local inflammation, and neurogenic hyperactivity [4]. Because these pathological networks are intertwined, the current clinical paradigm—spanning from conservative management and pharmacotherapy to neuromodulation and end‐stage surgery—often proves inadequate [1]. By targeting isolated pathways, these conventional modalities largely provide palliative symptom management rather than fundamentally modifying the disease course. Consequently, there is an urgent clinical need for comprehensive regenerative strategies capable of addressing the full complexity of the condition and restoring long‐term bladder homeostasis.

Stem cell therapy has emerged as a promising frontier in urological regenerative medicine, offering a potential solution to this therapeutic impasse. Following early translational successes in conditions such as erectile dysfunction [5] and stress urinary incontinence [6], its application in treating refractory bladder disorders has drawn increasing attention [7]. Growing evidence suggests that, beyond simply replacing damaged tissue through direct engraftment, the therapeutic efficacy of stem cells—particularly nonhematopoietic, multipotent mesenchymal stem/stromal cells (MSCs)—is largely driven by their pleiotropic paracrine activity [8, 9]. Through the release of a diverse secretome comprising various growth factors and cytokines, these cells can attenuate inflammatory responses, protect urothelial cells from apoptosis, and mitigate fibrotic remodeling [10, 11]. This multifaceted, multitarget mechanism is highly congruent with the complex pathology of IC/BPS, providing a strong biological rationale for sustained structural and functional recovery.

Despite this mechanistic promise, the clinical translation of stem cell therapy for IC/BPS remains in its infancy, with only a single pioneering human trial reported to date [12]. In this context, recent comprehensive reviews have highlighted the emerging, albeit limited, clinical landscape of regenerative medicine strategies (including MSCs, stromal vascular fraction, and platelet‐rich plasma) in human patients, underscoring the urgent need to standardize preclinical evidence to guide future trials [13]. A critical barrier to broader clinical application is the fragmented state of existing preclinical data. Although in vivo models are indispensable, the current literature exhibits substantial methodological heterogeneity. Variations in cell sources, delivery routes, and disease induction models complicate the objective evaluation of therapeutic efficacy. Without defining optimal treatment parameters from these foundational studies, the design of rigorous human trials remains severely hampered. To bridge this translational gap, we conducted a systematic review and meta‐analysis of preclinical studies evaluating stem cell therapy for IC/BPS. By quantitatively synthesizing efficacy outcomes and elucidating underlying mechanisms across diverse protocols, we aim to establish an evidence‐based framework to optimize interventional parameters and accelerate future clinical translation.

## 2. Materials and Methods

### 2.1. Study Design and Registration

This systematic review and meta‐analysis were conducted in accordance with the Preferred Reporting Items for Systematic Reviews and Meta‐Analyses (PRISMA) 2020 guidelines [14, 15]. The study protocol was prospectively registered in the PROSPERO database prior to initiating the literature search (ID: CRD420251151132).

### 2.2. Literature Search

A comprehensive search of PubMed, Embase, Web of Science, and the Cochrane Library was performed from database inception through July 8, 2025. The search strategy integrated core stem cell terms (e.g., “stem cell”, “mesenchymal stem cell”) with target condition descriptors (“interstitial cystitis”, “bladder pain syndrome”). This approach utilized a combination of Medical Subject Headings (MeSH), free‐text keywords, and Boolean operators (the complete search syntax for each database is detailed in Table S1). To maintain a high standard of evidence, the search was restricted to original, peer‐reviewed preclinical studies published as full‐length articles in English.

### 2.3. Eligibility Criteria

Eligibility criteria were predefined according to the PICOS framework: (1) Population (P): preclinical in vivo models of IC/BPS, regardless of animal species, sex, or the specific disease‐induction methods. (2) Intervention (I): experimental cohorts receiving stem cell monotherapy irrespective of stem cell type, dosage, or administration route. (3) Comparison (C): disease‐induced animals serving as controls, which received vehicle treatment or remained untreated. (4) Outcomes (O): quantitative assessments of bladder histopathology, urodynamic profiles and voiding patterns, or nociceptive behaviors. (5) Study design (S): controlled in vivo laboratory experiments.

Conversely, studies were excluded if they met any of the following criteria: (1) human clinical trials or purely in vitro investigations; (2) administration of combination therapies, cell‐free derivatives (e.g., isolated exosomes), or unpurified mixed populations (e.g., stromal vascular fraction); (3) absence of an appropriate disease‐induced control cohort; and (4) nonoriginal publications (e.g., reviews, editorials, and abstracts) or articles lacking extractable quantitative data.

### 2.4. Data Extraction

Two independent investigators (YL and XZ) screened the literature and extracted data (bibliographic details, model characteristics, treatment protocols, and quantitative outcomes) using a standardized template. A third investigator (KF) adjudicated any discrepancies. For outcomes measured at multiple time points, data from the final follow‐up were preferentially extracted to assess sustained therapeutic efficacy. However, in acute induction models prone to spontaneous resolution, values representing the maximal physiological response prior to natural recovery were recorded. When a single study included multiple experimental groups that shared a common control group, we divided the sample size of the shared control group equally among the treatment arms. However, in cases where this division would yield fractional control groups (e.g., *n* < 2) that destabilize continuous data models, we selectively included only the most clinically representative arms. In cases where original numerical data were not explicitly reported, graphical data were digitized using GetData Graph Digitizer (version 2.26, S. Fedorov, Moscow, Russia). Furthermore, to ensure statistical consistency for continuous outcomes, all standard error of the mean (SEM) values were mathematically converted to standard deviation (SD) prior to pooling.

### 2.5. Risk of Bias Assessment

Two independent investigators (TY and WT) assessed the methodological quality of the included studies using the SYRCLE risk‐of‐bias tool [16], which evaluates 10 bias domains across selection, performance, detection, attrition, and reporting biases. Each domain was assigned a judgment of “low”, “high”, or “unclear” risk. Discrepancies were resolved through consensus or via adjudication by a third senior investigator (KF).

### 2.6. Statistical Analysis

All statistical analyses were executed using Review Manager (RevMan) version 5.4. To account for the diverse measurement scales used across studies, we pooled continuous outcomes using the standardized mean difference (SMD) with 95% confidence intervals (CIs). Although our preregistered protocol initially proposed a fixed‐effect model for low heterogeneity (*I^2^* < 50%), we ultimately applied a random‐effects model across all data syntheses. This adjustment was made because a random‐effects model more conservatively and robustly accounts for the substantial inherent biological and methodological variations across preclinical in vivo studies. Heterogeneity was quantified via the *I^2^* statistic and evaluated using Cochran’s Q test (significance threshold *p*  < 0.05). To evaluate the robustness of the pooled effect sizes, we conducted sensitivity analyses using the leave‐one‐out method. Where substantial heterogeneity existed, prespecified subgroup analyses (based on cell type, dose, delivery route, transplant type, and induction model) were employed. Publication bias was assessed by visually inspecting funnel plots, specifically for core histopathological outcomes comprising 10 or more studies.

Furthermore, the overall certainty of evidence was qualitatively evaluated. Due to the exploratory nature of preclinical in vivo research, a formal GRADE framework was not strictly quantified; rather, confidence in the body of evidence was interpreted based on methodological rigor, effect size magnitude, and interstudy consistency. Within this qualitative analytical framework, bladder tissue‐restorative parameters (attenuation of mast cell infiltration, fibrosis, and apoptosis) were designated as the primary endpoints. Consequently, our comprehensive certainty assessment—integrating the SYRCLE risk of bias, statistical heterogeneity, and publication bias profiles—was strategically anchored to these core histopathological indicators. Conversely, for secondary outcomes (local inflammatory markers, urodynamic profiles, and nociceptive behaviors), certainty evaluation was primarily anchored to the SYRCLE framework and effect magnitude to maintain methodological focus.

## 3. Results

### 3.1. Study Selection and Search Outcomes

Our initial database search yielded 463 records (Web of Science: 216; Cochrane Library: 72; PubMed: 78; and Embase: 97). Following the removal of 123 duplicates, 340 unique citations underwent title and abstract screening, which eliminated 309 clearly irrelevant records. During the subsequent full‐text review of the remaining 31 candidates, 13 articles were excluded for failing to meet our predefined PICOS criteria. Ultimately, 18 preclinical studies [17–34] were incorporated into the final quantitative synthesis. The complete selection process is detailed in the PRISMA flow diagram (Figure 1).

**Figure 1:**
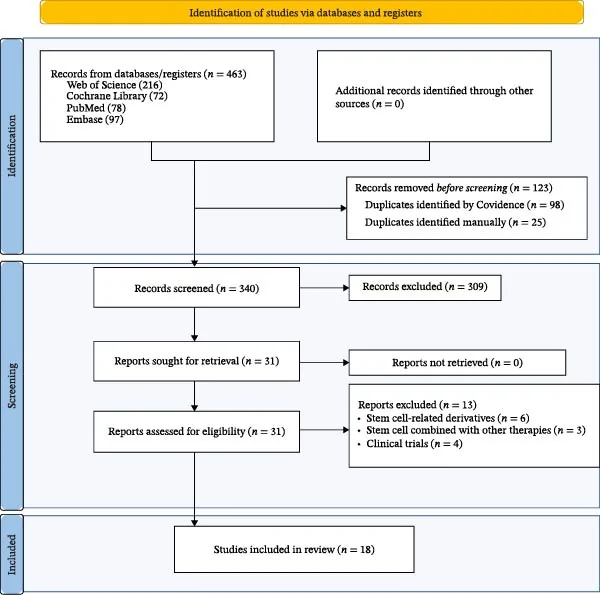
PRISMA flow diagram.

### 3.2. Study Characteristics

The characteristics of the 18 included studies [17–34], comprising 27 comparisons, are detailed in Tables 1 and 2. All preclinical investigations were conducted in East Asia (South Korea, *n* = 7; China, *n* = 6; and Japan, *n* = 5). Methodologically, the studies exhibited substantial heterogeneity in disease modeling. While rat models were employed exclusively, the induction protocols varied considerably, encompassing acute chemical injuries (hydrochloric acid [HCl]: *n* = 7 [17–19, 21, 22, 24, 26]; cyclophosphamide [CYP]: *n* = 4 [20, 23, 31, 32]) and chronic immunological or toxicological challenges, such as protamine sulfate (PS) and/or lipopolysaccharide (LPS) [25, 29, 30], ketamine (KC) [27, 34], and uroplakin II (UPKII) autoimmunity [33]. Additionally, one study utilized the Toll‐like receptor 7 (TLR7) agonist loxoribine (LX) to replicate a clinically relevant Hunner‐type IC (HIC) phenotype [28].

**Table 1:** Baseline characteristics of the included studies.

Study	Country	Species sex	Age weight	Method of induction	Outcomes
Furuta et al. [17]	Japan	F344 rats; Female	160–200 g	HCl	A:(1)(2)(4) B:(1)(3)(4)(6) C:(1)
Hendawy et al. [18]	Japan	SD rats; Female	8 weeks; 250–300 g	HCl	A:(1)(2)
Furuta et al. [19]	Japan	F344 rats; Female	100–140 g	HCl	A:(4) B:(1)(3)(4)(6)(9) C:(1)
Xu et al. [20]	China	SD rats; Female	200–250 g	CYP	A:(1)(4) B:(7)
Kim et al. [21]#1	Korea	Rats	NR	HCl	A:(1)(2)(3) B:(1)(2)(3)(4)(5)(6)(7)(8)
Kim et al. [21]#2	Korea	Rats	NR	HCl	A:(1)(2)(3) B:(1)(2)(3)(4)(5)(6)(7)(8)
Kim et al. [21]#3	Korea	Rats	NR	HCl	B:(1)(4)(5)(6)(7)(8)
Kim et al. [21]#4	Korea	Rats	NR	HCl	B:(1)(4)(5)(6)(7)(8)
Kim et al. [21]#5	Korea	Rats	NR	HCl	B:(1)(4)(5)(6)(7)(8)
Kim et al. [21]#6	Korea	Rats	NR	HCl	B:(1)(4)(5)(6)(7)(8)
Hirose et al. [22]	Japan	F344 rats; Female	120–140 g	HCl	A:(4)(5)(6) B:(1)(3)(7) C:(1)
Gao et al. [23]	China	SD rats; Female	180–210 g	CYP	B:(1)(2)(9)
Yu et al. [24]	Korea	SD rats; Female	10 weeks	HCl	A:(1)(2)(3) B:(3)(4)(5)(6)(7)(8)
Ryu et al. [25]#1	Korea	SD rats; Female	10 weeks	PS + LPS	A:(1)(3) B:(1)(2)(3)(5)(6)(7)(8)
Ryu et al. [25]#2	Korea	SD rats; Female	10 weeks	PS + LPS	A:(1)(3) B:(1)(2)(3)(5)(6)(7)(8)
Ryu et al. [25]#3	Korea	SD rats; Female	10 weeks	PS + LPS	A:(1)(3) B:(4)(5)(7)(8)
Song et al. [26]	Korea	SD rats; Female	10 weeks	HCl	B:(7)
Kim et al. [27]	Korea	SD rats; Female	10 weeks	KC	A:(1)(2)(3)(4) B:(4)(7)
Tabata et al. [28]	Japan	SD rats; Female	8 weeks	LX	B:(5)(9) C:(1)
Xiao et al. [29]	China	SD rats; Male/Female	4 months; 200–250 g	PS	B:(1)(2)(4)(5)(6)(7)
Li et al. [30]	China	SD rats; Female	200–230 g	PS + LPS	A:(1)(3)(4)(5) B:(1)(2)(5)(9)
Gao et al. [31]	China	SD rats; Female	200–220 g	CYP	B:(1)(2)(9)
Xie et al. [32]	China	SD rats; Female	200–250 g	CYP	A:(1)(4)(5)(6) B:(7)
Chung et al. [33]	Korea	SD rats; Female	8 weeks	UPKII	A:(1) B:(7)(9)
Lee et al. [34]#1	Korea	SD rats; Female	10 weeks	KC	A:(1)(2)(3) B:(2)(4)(7)
Lee et al. [34]#2	Korea	SD rats; Female	10 weeks	KC	A:(1)(2)(3) B:(2)(4)(7)
Lee et al. [34]#3	Korea	SD rats; Female	10 weeks	KC	A:(1)(2)(3) B:(2)(4)(7)

*Note:* Outcomes evaluated include: A. Histopathological and inflammatory outcomes: (1) mast cell infiltration, (2) fibrosis, (3) apoptosis, (4) TNF‐α, (5) IL‐6, (6) IL‐1β; B. Cystometric and voiding parameters: (1) basal pressure, (2) maximum pressure, (3) micturition pressure, (4) bladder capacity, (5) voided volume, (6) postvoid residual volume (PVR), (7) intercontraction interval (ICI), (8) nonvoiding contraction (NVC), (9) voiding frequency; C. Nociceptive behavior: (1) freezing behavior.

Abbreviations: CYP, cyclophosphamide; HCl, hydrochloric acid; KC, ketamine; LPS, lipopolysaccharide; LX, loxoribine; NR, not reported; PS, protamine sulfate; UPKII, uroplakin II.

**Table 2:** Intervention protocols of the included studies.

Study	Cell type	Transplant type	Cell number	Administration route	Vehicle
Furuta et al. [17]	ADSCs	Allogeneic	1 × 10^6^	BWI	PBS
Hendawy et al. [18]	ADSCs	Allogeneic	2 × 10^6^	BWI	PBS
Furuta et al. [19]	Muse cells	Xenogeneic (human)	2 × 10^4^	BWI	HBSS
Xu et al. [20]	hUC‐MSCs	Xenogeneic (human)	1 × 10^6^	TVI	PBS
Kim et al. [21]#1	M‐MSCs	Xenogeneic (human)	2.5 × 10^5^	BWI	None
Kim et al. [21]#2	M‐MSCs	Xenogeneic (human)	1 × 10^6^	BWI	None
Kim et al. [21]#3	BMSCs	Xenogeneic (human)	1 × 10^5^	BWI	None
Kim et al. [21]#4	BMSCs	Xenogeneic (human)	2.5 × 10^5^	BWI	None
Kim et al. [21]#5	M‐MSCs	Xenogeneic (human)	1 × 10^5^	BWI	None
Kim et al. [21]#6	M‐MSCs	Xenogeneic (human)	2.5 × 10^5^	BWI	None
Hirose et al. [22]	DPSCs	Xenogeneic (human)	2 × 10^6^	BWI	PBS
Gao et al. [23]	hUC‐MSCs	Xenogeneic (human)	8 × 10^5^	IT	PBS
Yu et al. [24]	M‐MSCs	Xenogeneic (human)	1 × 10^6^	BWI	PBS
Ryu et al. [25]#1	M‐MSCs	Xenogeneic (human)	2.5 × 10^5^	BWI	PBS
Ryu et al. [25]#2	M‐MSCs	Xenogeneic (human)	1 × 10^6^	BWI	PBS
Ryu et al. [25]#3	M‐MSCs	Xenogeneic (human)	1 × 10^6^	BWI	PBS
Song et al. [26]	hUC‐MSCs	Xenogeneic (human)	1 × 10^6^	BWI	PBS
Kim et al. [27]	hUC‐MSCs	Xenogeneic (human)	1 × 10^6^	BWI	PBS
Tabata et al. [28]	BMSCs	Allogeneic	1 × 10^6^	EJVI	DMEM
Xiao et al. [29]	BMSCs	Allogeneic	2 × 10^3^ (3 times/week for 4 weeks)	BWI	None
Li et al. [30]	USCs	Xenogeneic (human)	1.2 × 10^6^	Intravenously	None
Gao et al. [31]	hUC‐MSCs	Xenogeneic (human)	1 × 10^6^	IT	PBS
Xie et al. [32]	hUC‐MSCs	Xenogeneic (human)	1 × 10^6^	TVI	PBS
Chung et al. [33]	USCs	Xenogeneic (human)	1 × 10^5^	BWI	PBS
Lee et al. [34]#1	M‐MSCs	Xenogeneic (human)	2.5 × 10^5^	BWI	PBS
Lee et al. [34]#2	M‐MSCs	Xenogeneic (human)	5 × 10^5^	BWI	PBS
Lee et al. [34]#3	M‐MSCs	Xenogeneic (human)	1 × 10^6^	BWI	PBS

*Note:* For Xiao et al. [29], BMSCs were administered via multiple injections (10 μL per injection, 2 × 10^5^ cells/mL), three times a week for 4 weeks.

Abbreviations: ADSCs, adipose‐derived stem cells; BMSCs, bone marrow mesenchymal stem cells; BWI, bladder wall injection; DMEM, Dulbecco’s modified Eagle medium; DPSCs, dental pulp stem cells; EJVI, external jugular vein injection; HBSS, Hank’s balanced salt solution; hUC‐MSCs, human umbilical cord‐derived mesenchymal stem cells; IT, intrathecal injection; M‐MSCs, multipotent mesenchymal stem cells (human embryonic stem cell‐derived); Muse, multilineage‐differentiating stress enduring cells; NR, not reported; PBS, phosphate‐buffered saline; TVI, tail vein injection; USCs, urine‐derived stem cells.

Regarding stem cell therapeutic strategies, while our initial inclusion criteria broadly encompassed all stem cell types, the evaluated interventions were ultimately strictly limited to MSCs or cells exhibiting highly analogous nonhematopoietic, multipotent properties. A broad spectrum of these cell types was investigated—adipose‐derived stem cells (ADSCs), bone marrow mesenchymal stem cells (BMSCs), dental pulp stem cells (DPSCs), human umbilical cord‐derived mesenchymal stem cells (hUC‐MSCs), human embryonic stem cell (hESC)‐derived multipotent mesenchymal stem cells (M‐MSCs), multilineage‐differentiating stress enduring (Muse) cells, and urine‐derived stem cells (USCs)—with a predominant focus on human‐derived over rat‐derived sources (78% vs. 22%). Despite the diversity in cellular types, administration protocols remained remarkably consistent. Single‐dose interventions were utilized in nearly all studies (17/18, 94%), with dosages ranging from 2 × 10^4^ to 2 × 10^6^ cells. Notably, the widespread adoption of bladder wall injection (BWI, *n* = 12) and the frequent use of a dose of 1 × 10^6^ cells (*n* = 11) provided a solid methodological foundation for the subsequent stratified subgroup analyses. Ultimately, every included study reported at least one predefined quantitative outcome.

### 3.3. Risk of Bias Assessment

The internal validity of the 18 included preclinical studies was assessed using the SYRCLE risk‐of‐bias tool. Overall, the literature was characterized by incomplete methodological reporting, resulting in a high frequency of “unclear” risk judgments across multiple domains (Figure 2).

**Figure 2:**
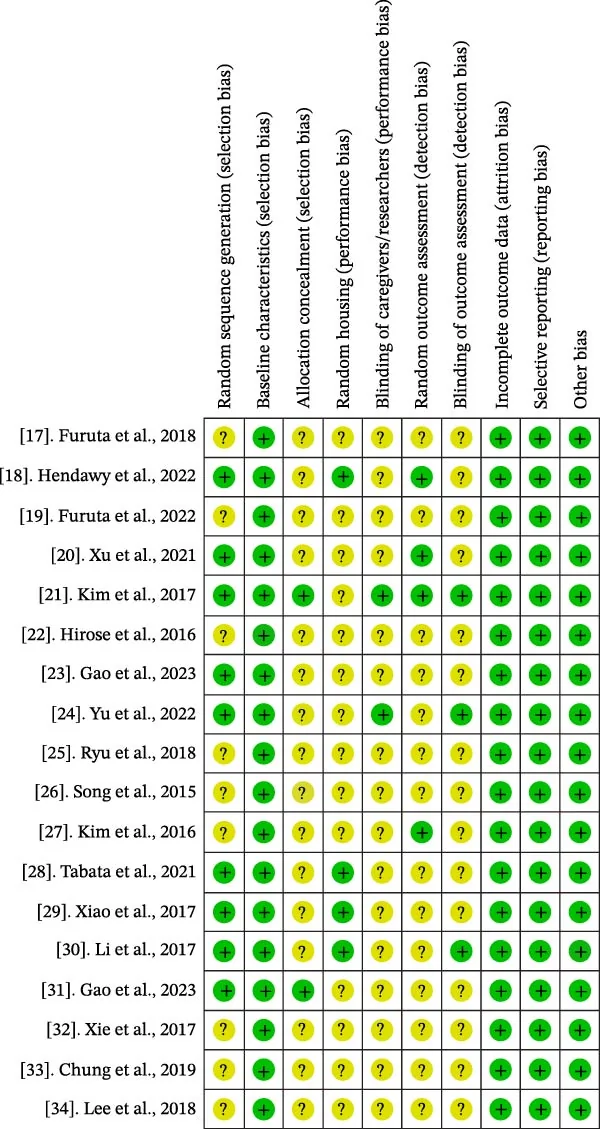
Risk of bias summary.

Although all studies successfully established baseline comparability, random sequence generation was explicitly mentioned in only half (9/18) [18, 20, 21, 23, 24, 28–31]. Merely two trials detailed the specific randomization method [23, 31], and only two ensured adequate allocation concealment [21, 31]. Blinding was similarly underreported: only three studies reported blinded outcome assessment, comprising two double‐blind designs [21, 24] and one single‐blind approach [30]. Other critical measures, such as random animal housing [18, 28–30] and randomized outcome assessment (e.g., specimen selection) [18, 20, 21, 27], were adequately described in only four studies each (22.2%). Notably, all studies demonstrated a universally low risk for both attrition and selective reporting biases. Collectively, these prevalent reporting limitations partially compromise the assessment of internal validity and hinder the reliable translation of these preclinical findings into clinical practice.

### 3.4. Meta‐Analysis of Histopathological and Tissue‐Restorative Effects

#### 3.4.1. Pooled Efficacy on Core Pathological Indices

Quantitative synthesis demonstrated consistent bladder tissue‐restorative effects of MSC therapy in preclinical IC/BPS models. Specifically, the interventions significantly attenuated mast cell infiltration [SMD = −2.42, 95% CI (−3.17, −1.66), *p*  < 0.00001, *I^2^* = 69%; *k* = 16] (Figure 3a), reduced collagen deposition to mitigate fibrosis [SMD = −2.13, 95% CI (−2.67, −1.59), *p*  < 0.00001, *I^2^* = 7%; *k* = 9] (Figure 3b), and inhibited urothelial apoptosis [SMD = −3.09, 95% CI (−3.94, −2.23), *p*  < 0.00001, *I^2^* = 40%; *k* = 11] (Figure 3c). Notably, while statistical heterogeneity was low to moderate for fibrosis (*I^2^* = 7%) and apoptosis (*I^2^* = 40%), substantial heterogeneity was observed in mast cell infiltration (*I^2^* = 69%). Nevertheless, the uniform direction of the treatment effects strongly supports the broader cytoprotective efficacy of MSC therapies.

**Figure 3:**
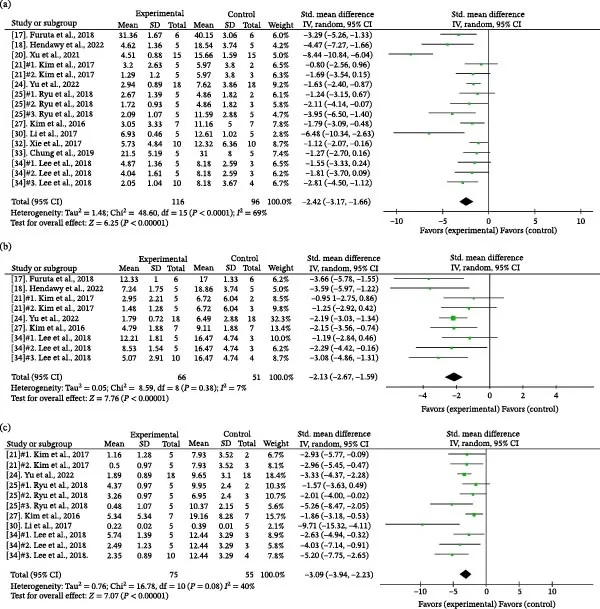
Forest plots for the effects of stem cell therapy on histopathological parameters: (a) mast cell infiltration, (b) collagen deposition, and (c) apoptosis.

#### 3.4.2. Subgroup Analyses

We performed prespecified subgroup analyses across the three primary pathological outcomes to explore potential sources of heterogeneity and evaluate the influence of different study designs (Table 3). Interestingly, therapeutic efficacy proved independent of the disease model, yielding consistent benefits across various IC/BPS induction methods (e.g., HCl, KC, PS + LPS) for all three core pathological outcomes (all subgroup difference *p* ≥ 0.42).

**Table 3:** Subgroup analysis of bladder histopathology.

Moderators	Trials (*n*)	SMD (95% CI)	Test for subgroup differences		Heterogeneity within subgroups	
			*p*‐value	*I* ^2^ (%)	*I* ^2^ (%)	*p*‐value
Mast cell infiltration	16 (212)	−2.42 [−3.17, −1.66]	—	—	—	—
Cell type	—	—	0.10	51.80	—	—
ADSCs	2 (22)	−3.68 [−5.29, −2.07]	—	—	0	0.50
M‐MSCs	9(106)	−1.77 [−2.28, −1.27]	—	—	0	0.65
hUC‐MSCs	3 (64)	−3.57 [−6.81, −0.33]	—	—	94	<0.00001
USCs	2 (20)	−3.56 [−8.63, 1.51]	—	—	84	0.01
Cell number	—	—	0.02	62.20	—	—
1 × 10^5^	1 (10)	−1.27 [−2.70, 0.16]	—	—	NA	NA
2.5 × 10^5^	3 (22)	−1.19 [−2.24, −0.14]	—	—	0	0.84
5 × 10^5^	1 (8)	−1.81 [−3.70, 0.09]	—	—	NA	NA
1 × 10^6^	9 (152)	−2.72 [−3.80, −1.65]	—	—	78	<0.0001
1.2 × 10^6^	1 (10)	−6.48 [−10.34, −2.63]	—	—	NA	NA
2 × 10^6^	1 (10)	−4.47 [−7.27, −1.66]	—	—	NA	NA
Administration route	—	—	0.23	31.70	—	—
BWI	13(152)	−1.87 [−2.31, −1.43]	—	—	1	0.43
IV	3 (60)	−5.23 [−10.65, 0.19]	—	—	94	<0.00001
Transplant type	—	—	0.12	58.50	—	—
Allogeneic	2 (22)	−3.68 [−5.29, −2.07]	—	—	0	0.50
Xenogeneic	14 (190)	−2.26 [−3.05, −1.46]	—	—	70	<0.0001
Method of induction	—	—	0.63	0	—	—
CYP	2 (50)	−4.69 [−11.87, 2.48]	—	—	97	<0.00001
HCl	5 (73)	−2.04 [−3.02, −1.05]	—	—	44	0.13
KC	4 (44)	−1.98 [−2.78, −1.17]	—	—	0	0.74
PS + LPS	4 (35)	−2.98 [−4.86, −1.09]	—	—	58	0.07
UPKII	1 (10)	−1.27 [−2.70, 0.16]	—	—	NA	NA
Fibrosis	7 (117)	−2.13 [−2.67, −1.59]	—	—	—	—
Cell type	—	—	0.14	49.3	—	—
ADSCs	2 (22)	−3.63 [−5.21, −2.05]	—	—	0	0.97
M‐MSCs	6 (81)	−1.93 [−2.51, −1.35]	—	—	0	0.46
hUC‐MSCs	1 (14)	−2.15 [−3.56, −0.74]	—	—	NA	NA
Cell number	—	—	0.21	34.5	—	—
2.5 × 10^5^	2 (15)	−1.08 [−2.30, 0.14]	—	—	0	0.85
5 × 10^5^	1 (8)	−2.29 [−4.42, −0.16]	—	—	NA	NA
1 × 10^6^	5 (84)	−2.28 [−2.88, −1.68]	—	—	0	0.41
2 × 10^6^	1 (10)	−3.59 [−5.97, −1.22]	—	—	NA	NA
Transplant type	—	—	0.05	74.1	—	—
Allogeneic	2 (22)	−3.63 [−5.21, −2.05]	—	—	0	0.97
Xenogeneic	7 (95)	−1.96 [−2.50, −1.42]	—	—	0	0.58
Method of induction	—	—	0.97	0	—	—
HCl	5 (73)	−2.16 [−3.06, −1.26]	—	—	36	0.18
KC	4 (44)	−2.13 [−2.97, −1.29]	—	—	0	0.50
Apoptosis	11 (130)	−3.09 [−3.94, −2.23]	—	—	—	—
Cell type	—	—	0.01	76.3	—	—
M‐MSCs	9 (106)	−3.13 [−3.82, −2.43]	—	—	4	0.41
hUC‐MSCs	1 (14)	−1.86 [−3.18, −0.53]	—	—	NA	NA
USCs	1 (10)	−9.71 [−15.32, −4.11]	—	—	NA	NA
Cell number	—	—	0.07	58.1	—	—
2.5 × 10^5^	3 (22)	−2.24 [−3.60, −0.89]	—	—	0	0.69
5 × 10^5^	1 (8)	−4.03 [−7.14, −0.91]	—	—	NA	NA
1 × 10^6^	6 (90)	−3.09 [−4.11, −2.07]	—	—	44	0.11
1.2 × 10^6^	1 (10)	−9.71 [−15.32, −4.11]	—	—	NA	NA
Administration route	—	—	0.02	82.1	—	—
BWI	10 (120)	−2.89 [−3.60, −2.18]	—	—	19	0.27
IV	1 (10)	−9.71 [−15.32, −4.11]	—	—	NA	NA
Method of induction	—	—	0.42	0	—	—
HCl	4 (65)	−2.79 [−3.54, −2.04]	—	—	0	0.40
KC	3 (30)	−3.85 [−5.42, −2.29]	—	—	7	0.34
PS + LPS	4 (35)	−3.71 [−6.30, −1.13]	—	—	70	0.02

Abbreviations: ADSCs, adipose‐derived stem cells; BMSCs, bone marrow mesenchymal stem cells; BWI, bladder wall injection; CI, confidence interval; CYP, cyclophosphamide; HCl, hydrochloric acid; hUC‐MSCs, human umbilical cord‐derived mesenchymal stem cells; KC, ketamine; LPS, lipopolysaccharide; M‐MSCs, multipotent mesenchymal stem cells (human embryonic stem cell‐derived); NA, not applicable; PS, protamine sulfate; SMD, standardized mean difference; UPKII, uroplakin II; USCs, urine‐derived stem cells.

Regarding cellular characteristics, no significant subgroup differences were observed in mitigating tissue fibrosis (*p* = 0.14) or mast cell infiltration (*p* = 0.10) across different cell types. Notably, mainstream cell types such as M‐MSCs demonstrated highly consistent therapeutic effects, exhibiting zero statistical heterogeneity (*I^2^* = 0%) across multiple indices. Although a significant subgroup difference was noted in apoptosis (*p* = 0.01), this was primarily driven by an extreme effect size from a single USC trial [30] characterized by an exceedingly wide 95% confidence interval [−15.32, −4.11]. In terms of transplant types, although xenogeneic transplantation—accounting for the vast majority of the statistical weight—effectively ameliorated all pathological measures, allogeneic transplantation exhibited a stronger effect in reversing chronic tissue fibrosis (SMD = −3.63 vs. −1.96; subgroup difference *p* = 0.05). Moreover, despite the limited number of trials, the allogeneic cohort demonstrated remarkable internal consistency (*I^2^* = 0%).

Beyond cell type, we evaluated dosing and administration strategies. Although certain lower or higher doses (e.g., 5 × 10^5^ or 2 × 10^6^) occasionally yielded larger numerical effect sizes, these observations predominantly originated from isolated trials (*k* = 1) with wide 95% confidence intervals. Conversely, the 1 × 10^6^ cells/rat cohort encompassed the vast majority of the cumulative sample size and demonstrated highly stable tissue repair (e.g., zero statistical heterogeneity in mitigating fibrosis). Similarly, the administration route played a crucial role in data stability. Localized BWI consistently produced significant tissue improvements with exceptionally low statistical heterogeneity (e.g., *I^2^* = 1% for mast cell infiltration and *I^2^* = 19% for apoptosis). Systemic intravenous (IV) administration, by contrast, introduced substantial statistical instability, characterized by severe heterogeneity (e.g., *I^2^* = 94% for mast cell infiltration) and a wide 95% confidence interval that crossed the line of no effect [−10.65, 0.19].

#### 3.4.3. Sensitivity Analysis and Publication Bias

Leave‐one‐out sensitivity analysis confirmed the robust stability of primary pathological outcomes (overall effect *p*  < 0.00001 across all omissions; Tables S2a–c). Crucially, this step identified potential sources of methodological heterogeneity, corroborating our earlier subgroup findings. For instance, omitting Xu et al. [20] significantly reduced the heterogeneity (*I^2^*) for mast cell infiltration from 69% to 30%. This study employed tail vein injection, indicating that IV delivery may introduce greater statistical instability than reliable localized BWI. Likewise, excluding Li et al. [30] lowered the heterogeneity in apoptosis from 40% to 19%. This trial utilized a unique methodological combination—USCs, a nonstandard dose (1.2 × 10^6^), and IV—generating an extreme, isolated effect size (SMD = −9.71) that deviated from mainstream protocols. Conversely, sensitivity analysis for fibrosis demonstrated remarkable consistency. The baseline heterogeneity was exceptionally low (*I^2^* = 7%) and remained minimal (*I^2^* ranging from 0% to 18%) across all individual study omissions. This unwavering stability further reinforces the highly reproducible antifibrotic efficacy of MSC therapies across diverse experimental conditions.

Visual inspection of the funnel plots for the primary pathological outcomes revealed that while the majority of studies clustered symmetrically around the pooled effect sizes (most notably for tissue fibrosis, Figure S1b), mild asymmetry was observed in other indices, characterized by a few small‐sample studies reporting exaggerated effect sizes (Figure S1). This observation suggests the potential presence of small‐study effects, consistent with the well‐documented vulnerability of preclinical in vivo models to publication bias. Importantly, the pronounced visual asymmetry in the apoptosis plot (Figure S1c) was primarily driven by the specific methodological outlier [30] previously identified in our sensitivity analysis, rather than a systemic publication flaw. Furthermore, we acknowledge the intrinsic limitations of assessing funnel plots with constrained study numbers (e.g., tissue fibrosis, *k* = 9). Despite these inherent limitations and the potential for publication bias, the substantial magnitude of the pooled effect sizes, coupled with the robust stability confirmed by our sensitivity analyses, strongly indicates that these regenerative benefits reflect genuine biological efficacy rather than mere publication artifacts.

### 3.5. Modulation of the Local Inflammatory Microenvironment

Beyond tissue restoration, MSC interventions significantly reshaped the local inflammatory microenvironment. Quantitative synthesis revealed a robust downregulation of key proinflammatory cytokines, specifically TNF‐α [SMD = −2.82, 95% CI (−4.08, −1.55), *p*  < 0.0001, *I^2^* = 78%; *k* = 7] (Figure S2a), IL‐6 [SMD = −1.69, 95% CI (−2.45, −0.93), *p*  < 0.0001, *I^2^* = 0%; *k* = 3] (Figure S2b), and IL‐1β [SMD = −1.48, 95% CI (−2.29, −0.67), *p* = 0.0004, *I^2^* = 0%; *k* = 2] (Figure S2c).

### 3.6. Improvement of Urodynamic Profiles and Voiding Patterns

We performed a comprehensive meta‐analysis of nine urodynamic parameters to evaluate the effects of MSC transplantation on bladder function. The results demonstrated substantial functional recovery across several domains.

*Intravesical pressure dynamics:* MSC therapy successfully lowered both basal pressure [SMD = −0.95, 95% CI (−1.53, −0.38), *p* = 0.001, *I^2^* = 53%; *k* = 15] (Figure S3a) and maximum pressure [SMD = −0.92, 95% CI (−1.59, −0.25), *p* = 0.007, *I^2^* = 50%; *k* = 11] (Figure S3b). However, micturition pressure showed no statistically significant change [SMD = 0.61, 95% CI (−0.58, 1.81), *p* = 0.31, *I^2^* = 78%; *k* = 8] (Figure S3c).

*Bladder storage:* The treatment expanded overall bladder capacity [SMD = 2.69, 95% CI (1.90, 3.48), *p*  < 0.00001, *I^2^* = 62%; *k* = 15] (Figure S3d) and concurrently increased voided volume [SMD = 2.71, 95% CI (1.58, 3.83), *p*  < 0.00001, *I^2^* = 72%; *k* = 13] (Figure S3e). Notably, while storage capacity improved, it was accompanied by a marginal increase in postvoid residual volume (PVR) [SMD = 0.79, 95% CI (0.05, 1.53), *p* = 0.04, *I^2^* = 66%; *k* = 12] (Figure S3f).

*Micturition patterns and detrusor stability:* MSC therapy normalized micturition patterns by prolonging the intercontraction interval (ICI) [SMD = 2.39, 95% CI (1.56, 3.23), *p*  < 0.00001, *I^2^* = 79%; *k* = 20] (Figure S3g) and suppressing nonvoiding contractions (NVCs) [SMD = −1.56, 95% CI (−2.34, −0.78), *p*  < 0.0001, *I^2^* = 52%; *k* = 10] (Figure S3h). These urodynamic improvements translated into functional recoveries that closely correlate with clinical symptom relief, as evidenced by a significant reduction in urinary frequency [SMD = −1.83, 95% CI (−3.41, −0.24), *p* = 0.02, *I^2^* = 82%; *k* = 6] (Figure S3i).

### 3.7. Attenuation of Nociceptive Behaviors

MSC interventions effectively mitigated visceral nociception, as evidenced by a pronounced reduction in freezing behavior [SMD = −3.19, 95% CI (−4.11, −2.26), *p*  < 0.00001, *I^2^* = 0%; *k* = 4] (Figure S4). The substantial effect size, coupled with the absence of observed statistical heterogeneity, indicates a highly consistent and potent antinociceptive benefit across the included preclinical models.

### 3.8. Certainty of Evidence

Based on our qualitative integration of methodological domains, the overall certainty of the evidence is considered moderate. The certainty is universally downgraded by the prevalent “unclear” risk of bias across several SYRCLE domains for all outcomes (histopathological, inflammatory, urodynamic, and behavioral). Specifically, the lack of rigorous reporting on critical safeguards—such as allocation concealment and blinding—introduces methodological uncertainty and a potential risk of overestimating the intervention effect sizes. Consequently, although the substantial magnitude of the pooled effect sizes and the uniform direction of therapeutic benefits foster high confidence in biological plausibility and directional efficacy, the precise magnitude of these pooled effects remains uncertain and should be interpreted as indicative trends.

## 4. Discussion

To the best of our knowledge, this study represents the first systematic review and meta‐analysis synthesizing histopathological, urodynamic, and nociceptive behavioral data regarding preclinical stem cell therapies—specifically those utilizing MSCs—for IC/BPS. Although the pleiotropic regenerative and immunomodulatory effects of MSCs are theoretically well‐documented, our pooled data provide a preliminary statistical foundation supporting the manifestation of these biological mechanisms into quantifiable therapeutic benefits in animal models. By targeting various pathways of the multifactorial IC/BPS cascade, MSC‐based interventions appear to support structural repair and functional recovery in animal models, suggesting promising disease‐modifying potential. Ultimately, despite inherent methodological heterogeneity, our analysis provides encouraging evidence that stem cell therapy offers a promising and effective regenerative strategy for IC/BPS that warrants further clinical translation.

### 4.1. Pleiotropic Mechanisms of Action: From Microenvironmental Remodeling to Neuromodulation

Our quantitative findings offer a clear picture: MSC interventions significantly reduce mast cell infiltration and downregulate key proinflammatory cytokines (TNF‐α, IL‐6, and IL‐1β). In alignment with these findings, existing mechanistic studies elucidate that MSCs suppress mast cell chemotaxis and degranulation through paracrine signaling, effectively attenuating the release of inflammatory mediators like histamine and TNF‐α [20]. By reshaping the immune microenvironment in this way, MSCs lay the essential physiological groundwork for restoring bladder structure and function. Operating concurrently within this improved milieu, MSCs exert antifibrotic effects via dual mechanisms—which mechanistically corroborates the significant reduction in collagen deposition observed in our meta‐analysis. On one hand, by downregulating the core profibrotic TGF‐β/Smad signaling axis, MSCs suppress epithelial‐mesenchymal transition (EMT) and the abnormal deposition of extracellular matrix (ECM) [17, 27]. On the other hand, extrapolating from findings in other organ fibrosis models, they likely promote the active breakdown of existing fibrosis by rebalancing the local matrix metalloproteinase/tissue inhibitor of metalloproteinases (MMP/TIMP) enzyme ratio [35, 36].

Beyond clearing adverse pathological events, a critical dimension of their disease‐modifying capability lies in the reconstruction of the damaged urothelium. In vivo cell tracking reveals that engrafted cells can home to the injured tissue and undergo differentiation to reconstitute the bladder urothelial barrier [21, 22, 24, 26]. Synergistically, by activating the Wnt/β‐catenin‐EGF signaling pathways, MSCs effectively mobilize the host’s own endogenous progenitor cells—which are typically rendered quiescent by severe pathological stimuli—driving native tissue regeneration more comprehensively [26]. Crucially, these preclinical mechanisms—specifically the recovery of the urothelial barrier and the remodeling of the hollow organ’s tissue—closely align with emerging molecular paradigms for human bladder interstitial regeneration, thereby establishing a plausible bench‐to‐bedside parallel [13].

While structural reconstruction restores the physical barrier, it cannot fully explain the profound attenuation of nociceptive freezing behavior observed in our meta‐analysis. Instead, this behavioral improvement points directly to another critical dimension of MSC efficacy: their potent neuromodulatory capacity. Peripherally, by alleviating the pathological spatial clustering of mast cells around afferent nerve fibers, they effectively disrupt the detrimental crosstalk between local inflammation and pain signaling [21]. Centrally, MSCs ameliorate spinal dorsal horn (SDH) sensitization. They downregulate c‐Fos expression to attenuate neuronal excitability, while simultaneously rescuing the suppressed Sirt1/Nrf2/HO‐1 axis to scavenge reactive oxygen species (ROS) and suppress proinflammatory glial activation [19, 23].

The convergence of these pleiotropic mechanisms fundamentally explains one of the most notable findings from our subgroup analyses: the “model‐agnostic” nature of therapeutic efficacy. Rather than merely counteracting specific initial triggers, MSCs systematically intervene in the shared downstream pathological cascades of IC/BPS. These broad‐spectrum effects align well with the etiological heterogeneity of the disease, suggesting the potential of MSC‐based therapies to address multiple pathological pathways rather than providing mere targeted symptomatic relief, thereby offering a promising approach to remodel the complex IC/BPS pathological network in preclinical settings.

### 4.2. Urodynamic Modulation and Voiding Pattern Restoration: Efficacy and Safety Profile of MSC Intervention

Bladder hypersensitivity and diminished capacity are key drivers of the severe urinary urgency and frequency in IC/BPS [37]. Our meta‐analysis demonstrates that MSC transplantation significantly mitigates these functional deficits.

Notably, while storage capacity improved, it was accompanied by an increase in PVR (SMD = 0.79). Although elevated PVR typically raises concerns about detrusor underactivity [38], this change must be evaluated alongside overall voiding patterns. Pathological urinary retention would inherently compromise voiding intervals and exacerbate voiding frequency. Conversely, the robust reduction in voiding frequency (SMD = −1.83) and prolonged ICI (SMD = 2.39) functionally demonstrate that the expanded bladder reservoir was successfully emptied. Thus, this PVR elevation likely represents a nonpathological adaptation to an expanded bladder rather than true experimental urinary retention [39]. However, as a critical limitation in the current literature, none of the included studies explicitly evaluated the detrusor contractility index (DCI) or performed targeted smooth muscle histology to definitively rule out subclinical detrusor underactivity. Therefore, this potential risk of PVR elevation still cannot be totally overlooked. Future chronological studies should not only monitor the long‐term persistence of PVR but also explicitly incorporate DCI measurements and detrusor histological analyses to thoroughly confirm functional integrity.

Furthermore, although basal and maximum pressures were significantly attenuated, micturition pressure remained statistically unaltered. Because maximum pressure in cystitis models often reflects pathological, high‐amplitude spasms during filling, its attenuation—congruent with the suppressed NVC frequency (SMD = −1.56)—likely indicates a targeted reduction in storage‐phase detrusor overactivity [40]. Crucially, the preservation of micturition pressure highlights a potential safety advantage over conventional anticholinergics or botulinum toxin A (BoNT‐A). While these agents alleviate spasms via nonselective detrusor suppression and carry retention risks in vivo [41], MSC therapy appears to mitigate the pathological high‐tension state without compromising the physiological detrusor contractility required for active voiding. This balanced urodynamic modulation underscores its translational potential in restoring lower urinary tract homeostasis.

### 4.3. Translational Roadmap: Optimizing Cellular Parameters for Clinical Efficacy

Our subgroup analyses provide a crucial translational roadmap for optimizing MSC interventions, which can be logically delineated into four core pillars.

#### 4.3.1. Cell Type: From Benchmark Establishment to Precision Medicine

In the context of cell type, M‐MSCs constitute the prevailing backbone of preclinical IC/BPS research. Beyond their substantial sample size, quantitative synthesis reveals that M‐MSCs exhibit highly consistent interstudy effects, demonstrating zero statistical heterogeneity (*I^2^* = 0%) across multiple pathological indices. This is mechanistically corroborated by their stable in situ engraftment and prolonged survival [21, 22, 24]. Crucially, the pioneering first‐in‐human IC/BPS trial employed M‐MSCs, reporting encouraging safety and preliminary efficacy profiles in alleviating pelvic pain and repairing Hunner lesions [12]. This convergence of highly stable preclinical data and early‐phase clinical observations solidifies the candidate status of M‐MSCs as a promising reference benchmark against which future experimental therapies may be evaluated.

Furthermore, specific cell types may offer targeted pathophysiological advantages. For instance, ADSCs exhibit a potent antifibrotic capacity (potentially driven by hepatocyte growth factor (HGF) secretion and epigenetic miRNA modulation) [42, 43], whereas USCs appear to provide targeted antiapoptotic urothelial protection (likely mediated by PI3K/AKT pathway activation) [44]. While these cell‐type‐specific peaks remain preliminary due to limited sample sizes in current subgroups, they highlight a compelling avenue for future precision medicine. Rationally tailoring cell types to the predominant pathophysiological endotype of individual IC/BPS patients—such as prioritizing USCs for severe urothelial denudation—represents a promising mechanism‐driven regenerative strategy that deserves further research.

#### 4.3.2. Dosage: Defining the Evidence‐Based Therapeutic Window

Rather than establishing a strictly linear dose–response relationship, our analysis highlights 1 × 10^6^ cells per rat as the evidence‐based dosage currently available. Unrestricted dose escalation is biologically precluded by safety concerns as excessive cell loads in the confined bladder wall may precipitate microvascular embolism or localized immune hyper‐reactivity [45, 46]. Therefore, safely translating this evidence‐based reference dosage into human clinical parameters is of critical importance.

Because MSCs are delivered locally into the bladder wall in most preclinical studies, standard body surface area (BSA)‐based allometric scaling is pharmacologically inappropriate [47]. Instead, scaling based on the target‐organ mucosal surface area serves as a more biologically relevant approach [48]. A schematic diagram illustrating this translation methodology is provided in Figure 4. For a standard rat bladder distended with 0.2 mL of fluid, the empirical mucosal surface area is ~2.25 cm^2^ [49], yielding a therapeutic density of 4.44 × 10^5^ cells/cm^2^ for the 1 × 10^6^ cells dose (calculated as 1 × 10^6^ cells ÷ 2.25 cm^2^). Assuming a spherical model [48], a partially distended human bladder during clinical cystoscopy (≈ 100 mL) has a surface area of ≈104 cm^2^. This translates to a theoretical human equivalent dose (HED) of ~4.6 × 10^7^ cells (calculated as 4.44 × 10^5^ cells/cm^2^ × 104 cm^2^).

**Figure 4:**
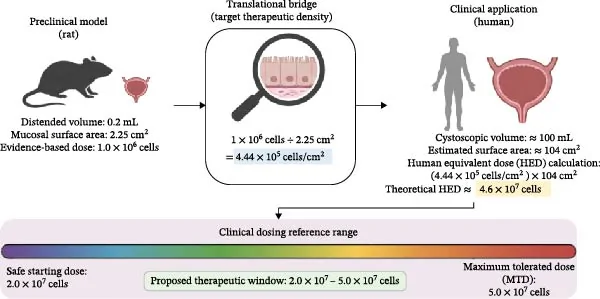
Schematic diagram of the mucosal surface area‐based allometric scaling framework: translation from the preclinical rat model to the theoretical human equivalent dose (HED) and proposed clinical therapeutic window.

This surface‐area‐based theoretical peak (4.6 × 10^7^ cells) closely matches the empirical maximum tolerated dose (MTD) of 5.0 × 10^7^ cells established in the only clinical trial of MSCs for IC/BPS to date [12]. This biological consistency cautions against oversimplified scaling purely by total organ volume (yielding > 2.0 × 10^8^ cells, which exceeds local tissue tolerance). By integrating the clinical safe starting dose (2.0 × 10^7^ cells) [12] as the active lower bound and the safety‐constrained MTD (5.0 × 10^7^ cells) as the upper ceiling, we propose the tailored range of 2.0 × 10^7^ to 5.0 × 10^7^ cells as a theoretically scaled, preliminary reference range to guide future clinical investigation.

#### 4.3.3. Administration Route: Localized BWI Versus Systemic IV

Beyond cellular parameters, the chosen delivery route profoundly influences the observed preclinical efficacy. Our analysis identifies localized BWI as a more reliable and reproducible delivery route compared to IV infusion. The statistical instability of the IV route finds a mechanistic explanation in the well‐documented “pulmonary first‐pass effect,” wherein intravenously administered MSCs are predominantly entrapped in the pulmonary capillary bed, rendering specific homing to the bladder highly stochastic [50, 51]. Notably, this pulmonary entrapment is not merely a passive filtration event but may also trigger phenotypic alterations in the entrapped cells, further attenuating their therapeutic payload [52, 53]. While IV infusion offers procedural convenience, its pharmacokinetic disadvantage in bladder targeting necessitates either substantial dose escalation (with attendant safety concerns) or adjunctive homing‐enhancement strategies. Conversely, BWI circumvents systemic circulation, optimizing localized cell retention and sustained therapeutic action directly at the injured bladder. Given that cystoscopy‐guided intravesical injections are already established in routine urological practice, BWI presents a highly feasible, safe, and readily translatable MSC delivery route for future human trials.

#### 4.3.4. Host Immune Microenvironment: Accounting for Translational Attenuation

Finally, interpreting the therapeutic efficacy captured in this meta‐analysis requires accounting for the host immune microenvironment—specifically, the translational attenuation imposed by xenogeneic immune recognition. Although xenogeneic models form the current preclinical backbone, interspecies antigenic differences inevitably provoke mild immune clearance, blunting the donor cells’ engraftment half‐life and paracrine output [52, 54]. This fundamental biological constraint provides a mechanistic explanation for the statistical superiority of allogeneic over xenogeneic models observed in our subgroup analysis. By bypassing this xenogeneic immune barrier, future autologous or human leukocyte antigen (HLA)‐matched allogeneic therapies in humans may yield prolonged cellular survival and enhanced reparative benefits [55]. However, whether these human therapies will substantially exceed the baseline efficacy captured in current preclinical models remains to be determined as the potential immunologic advantages must be weighed against the chronic, multifactorial, and highly treatment‐resistant nature of human IC/BPS.

### 4.4. Limitations of the Study

Several limitations inherent to the current preclinical landscape warrant acknowledgment. First, the majority of included studies exhibited an unclear risk of bias due to insufficient reporting on critical methodological safeguards, which substantially increases the risk of overestimated effect sizes and represents the primary factor downgrading our confidence in the precise quantitative findings. To address this reporting deficiency in line with the ARRIVE guidelines [56], future studies should prioritize transparent reporting of (1) explicit randomization and allocation concealment mechanisms, (2) distinct blinding protocols for animal caregivers, intervention administrators, and outcome assessors, and (3) a priori sample size justifications. Furthermore, we have compiled a concrete, actionable checklist of minimum reporting requirements in Table 4. Second, mild funnel plot asymmetry suggests potential small‐study effects and a publication bias favoring positive findings. Although the substantial magnitude of our pooled effects indicates genuine biological efficacy, partial inflation from this bias cannot be entirely dismissed. Third, profound methodological diversity in cell types, dosages, delivery routes, and induction models drove residual statistical heterogeneity. This precludes the establishment of a definitive “intervention‐model matrix” to perfectly match specific protocols with distinct pathological phenotypes. To mitigate this translational barrier, future studies should prioritize standardized, head‐to‐head comparative designs that directly contrast multiple cell types, dosages, or delivery routes within a single experimental framework. Lastly, the artificially induced rodent models predominantly simulate acute or subacute localized bladder inflammation [57]. While indispensable, they cannot fully replicate the long‐term, multifactorial progression of human IC/BPS, particularly its associated systemic features such as central nervous system sensitization and psychological comorbidities [57]. Consequently, although our findings demonstrate promising therapeutic potential, the translation of these preclinical results to human patients must be approached with caution.

**Table 4:** Summary checklist of minimum reporting recommendations for preclinical studies.

Item	Specific reporting requirement	Primary methodological domain	Rationale for recommendation
1. Randomization and allocation concealment	• Specify the exact method of random sequence generation (e.g., random number table and computer‐generated algorithm). • Describe the concealment procedure (e.g., sequentially numbered opaque sealed envelopes) to ensure allocation is unpredictable.	Selection bias (SYRCLE domains 1–3)	Prevents systematic imbalances in baseline characteristics and ensures group comparability beyond the mere statement of being “randomized”.

2. Blinding protocols	• Clearly report who was blinded: animal caregivers, intervention administrators, and/or outcome assessors. • If blinding was not feasible, explicitly state the reasons and describe any measures taken to minimize detection bias.	Performance and detection bias (SYRCLE domains 4–7)	Reduces subjective influence on intervention administration and outcome measurement, preventing the overestimation of treatment effects.

3. Sample size justification	• Provide a priori sample size calculation based on a defined effect size, alpha level (usually 0.05), and statistical power (commonly ≥80%). • Specify the statistical method used for the calculation.	Statistical precision (ARRIVE guidelines item 2)	Ensures adequate statistical power to detect true differences. Minimizes false‐negative errors and prevents unjustifiably large effect estimates typical of underpowered studies.

### 4.5. Frontiers in Preclinical Research: Toward Next‐Generation Therapies

To address these inherent limitations and accelerate clinical translation, the preclinical landscape is rapidly shifting toward next‐generation multimodal strategies. Rather than relying solely on generalized cell monotherapies, integrating MSCs with pharmacological agents (e.g., pentosan polysulfate sodium, *N*‐acetylcysteine, and melatonin) [58–60] demonstrates marked synergistic advantages. These combinations magnify therapeutic outcomes while significantly reducing the required cellular payload, thereby mitigating dose‐related risks. This is consistent with recent molecular frameworks suggesting that combining MSCs or autologous biological products with glycosaminoglycans, such as hyaluronic acid, can accelerate epithelialization, modulate the local immune response, and optimize connective tissue remodeling in the bladder wall [13]. Concurrently, targeted genetic engineering provides a precise mechanism to bolster the “survival‐engraftment‐repair” axis. Emerging evidence indicates that targeting key regulatory pathways—such as upregulating *FOS* and *CDK1* to promote stable tissue integration [24] or overexpressing HO‐1 to enhance stress‐resistant survival [31]—may enhance the capacity of transplanted cells to attenuate histopathological remodeling and alleviate IC/BPS‐associated visceral pain in preclinical models.

Furthermore, the shift toward cell‐free regenerative medicine represents a compelling strategy to circumvent the aforementioned xenogeneic immune barriers. MSC‐derived conditioned media (CM) and extracellular vesicles (EVs) have emerged as potent alternatives as they effectively encapsulate the paracrine potency of MSCs while fundamentally bypassing the immunogenic and tumorigenic risks associated with live‐cell engraftment [54, 61]. Given their proven efficacy in IC/BPS models [62, 63], cell‐free therapies pave a highly promising pathway to bridge the current translational gaps. Looking forward, the convergence of optimized cell‐processing technologies with interdisciplinary bioengineering promises a critical transition from generalized cell transplantation to precision regenerative medicine, offering more potent, targeted, and clinically accessible solutions for IC/BPS.

## 5. Conclusion

In conclusion, this comprehensive meta‐analysis highlights stem cell therapy—specifically the application of MSCs—as a promising intervention with potential disease‐modifying properties in preclinical models of IC/BPS, capable of ameliorating histopathology, supporting urodynamic homeostasis, and alleviating visceral pain. These regenerative benefits appear to be driven by a multitarget remodeling of the bladder microenvironment. Crucially, our findings provide a practical translational reference for future trial designs. Based on the most robustly supported preclinical rodent dose (1 × 10^6^ cells) and theoretical allometric scaling, we suggest a preliminary human therapeutic window of 2.0 × 10^7^ to 5.0 × 10^7^ cells, preferentially administered via localized BWI. Furthermore, while M‐MSCs currently represent the most comprehensively validated reference cell type, ADSCs and USCs present compelling avenues for phenotype‐specific precision medicine. Recognizing interspecies translational attenuation, bypassing xenogeneic immune barriers may hold the potential to enhance these regenerative benefits in human applications, although this must be rigorously evaluated against the complex nature of human IC/BPS. Ultimately, as the field evolves toward next‐generation cell‐free and multimodal strategies, this study establishes a critical foundational framework to help advance stem cell therapy (predominantly represented by MSCs) from preclinical concepts to rigorous clinical validation.

## Author Contributions

Junchao Wu conceived and designed the study. Yuanzhi Li and Xiang Zhao performed the literature search and data curation. Huitao Wang and Wu Tang performed formal analysis and risk of bias assessment. Tao Yang wrote the original draft. Kewei Fang acquired the funding. All authors contributed to writing – review and editing.

## Funding

This work was supported by the International Joint R&D Center for Key Technologies in Urological Diagnosis and Treatment of Yunnan Province (Grant 202403AP140016); the Zhang Yaoguang Expert Workstation of Yunnan Province (Grant 202405AF140058); and the Medical and Health Talents Project of the “Xingdian Talent Support Plan” in Yunnan Province (Grant XDYC‐YLWS‐2024‐0024).

## Disclosure

All authors have read and approved the final manuscript.

## Conflicts of Interest

The authors declare no conflicts of interest.

## Supporting Information

Additional supporting information can be found online in the Supporting Information section.

## Supporting information


**(e.g., Table S2, Figure S3)Supporting Information** The following Supporting Information are available for this article: Table S1: Search strategy (Date of search for all databases: 2025‐07‐08). Table S2: Sensitivity analyses of: (a) mast cell infiltration, (b) collagen deposition, and (c) apoptosis. Figure S1: Funnel plots evaluating publication bias. Assessment of publication bias for the meta‐analysis of (a) mast cell infiltration, (b) collagen deposition, and (c) urothelial apoptosis. Figure S2: Forest plots for the effects of stem cell therapy on proinflammatory cytokines: (a) TNF‐α, (b) IL‐6, and (c) IL‐1β. Figure S3: Forest plots for the effects of stem cell therapy on urodynamic parameters: (a) basal pressure, (b) maximum pressure, (c) micturition pressure, (d) bladder capacity, (e) voided volume, (f) postvoid residual volume, (g) intercontraction interval, (h) nonvoiding contractions, and (i) urinary frequency. Figure S4: Forest plots for the effects of stem cell therapy on nociceptive behaviors (freezing behavior).

## Data Availability

Study data are provided in the paper and Supporting Information files. Further information is available from the corresponding author upon reasonable request.
