# Supplementary material for: Efficacy of Stem Cell Therapy for Interstitial Cystitis/Bladder Pain Syndrome: A Systematic Review and Meta‐Analysis of Preclinical Studies
Source: Stem Cells Int. 2026 Oct 25;2026:4556842. doi: 10.1155/sci/4556842 (PMC13695852; doi:10.1155/sci/4556842)
Supplement: Supplementary file 1 [file SCI-2026-4556842-s001.zip › 4556842.f1/Table S1.docx]

**Table S1 Search strategy (Date of search for all databases: 2025-07-08)**

| **Database** | **Search Strategy Outline** | **Results** |
| --- | --- | --- |
| Web of Science (Clarivate) | (TS=(Stem Cells) OR AB=(Stem Cells OR stem cell OR mesenchymal stem cell OR mesenchymal stromal cell OR muse cell OR multilineage differentiating stress enduring cell OR progenitor cells OR cell therapy OR cell treatment OR cell transplantation OR cell transplant)) AND (TS=(Cystitis, Interstitial) OR AB=(Cystitis, Interstitial OR interstitial cystitis OR bladder pain syndrome)) | 216 |
| Embase (Elsevier) | #1 'stem cells':ab,ti OR 'stem cell':ab,ti OR 'mesenchymal stem cell':ab,ti OR 'mesenchymal stromal cell':ab,ti OR 'muse cell':ab,ti OR 'multilineage differentiating stress enduring cell':ab,ti OR 'progenitor cells':ab,ti OR 'cell  therapy':ab,ti OR 'cell treatment':ab,ti OR 'cell transplantation':ab,ti OR 'cell transplant':ab,ti 677,972  #2 'cystitis, interstitial':ab,ti OR 'interstitial cystitis':ab,ti OR 'bladder pain syndrome':ab,ti 6978  #3 #1 AND #2 97 | 97 |
| PubMed (NLM) | #1 ("stem cells"[MeSH Terms] OR "stem cells"[Title/Abstract] OR "stem cell"[Title/Abstract] OR "Mesenchymal stem cell"[Title/Abstract] OR "mesenchymal stromal cell"[Title/Abstract] OR "Muse cell"[Title/Abstract] OR "multilineage differentiating stress enduring cell"[Title/Abstract] OR "Progenitor cells"[Title/Abstract] OR "cell therapy"[Title/Abstract] OR "cell treatment"[Title/Abstract] OR "cell transplantation"[Title/Abstract] OR "cell Transplant"[Title/Abstract]) 538,428  #2 ("cystitis, interstitial"[MeSH Terms] OR "Cystitis,Interstitial"[Title/Abstract] OR "Interstitial cystitis"[Title/Abstract] OR "Bladder Pain syndrome"[Title/Abstract]) 4573  #3 #1 AND #2 78 | 78 |
| Cochrane Library (Wiley) | #1 MeSH descriptor: [Stem Cells] explode all trees 1332  #2 (Stem Cells or stem cell or mesenchymal stem cell or mesenchymal stromal cell or muse cell or multilineage differentiating stress enduring cell or progenitor cells or cell therapy or cell treatment or cell transplantation or cell transplant):ti,ab,kw (Word variations have been searched) 139215  #3 #1 or #2 139281  #4 MeSH descriptor: [Cystitis, Interstitial] explode all trees 263  #5 (Cystitis, Interstitial or interstitial cystitis or bladder pain syndrome):ti,ab,kw (Word variations have been searched) 901  #6 #4 or #5 901  #7 #3 and #6 72 | 72 |
