# Supplementary material for: Efficacy of Stem Cell Therapy for Interstitial Cystitis/Bladder Pain Syndrome: A Systematic Review and Meta‐Analysis of Preclinical Studies
Source: Stem Cells Int. 2026 Oct 25;2026:4556842. doi: 10.1155/sci/4556842 (PMC13695852; doi:10.1155/sci/4556842)
Supplement: Supplementary file 1 [file SCI-2026-4556842-s001.zip › 4556842.f1/Table S2 (revised version).docx]

**Table S2a Sensitivity analyses of Mast cell infiltration**

| Omitted study | SMD (95% CI) | ***p***-value  for Overall Effect | Heterogeneity  ***I²*** (%) | Heterogeneity  ***p***-value | Impact  Assessment |
| --- | --- | --- | --- | --- | --- |
| None | -2.42 [-3.17, -1.66] | <0.00001 | 69 | <0.0001 | Baseline |
| [17]. Furuta et al., 2018 | -2.36 [-3.15, -1.58] | <0.00001 | 70 | <0.0001 | Robust |
| [18]. Hendawy et al., 2022 | -2.32 [-3.08, -1.56] | <0.00001 | 69 | <0.0001 | Robust |
| **[20]. Xu et al., 2021** | **-1.91 [-2.41, -1.41]** | **<0.00001** | **30** | **0.13** | **Significantly reduced heterogeneity** |
| [21]#1. Kim et al., 2017 | -2.54 [-3.33, -1.74] | <0.00001 | 70 | <0.0001 | Robust |
| [21]#2. Kim et al., 2017 | -2.48 [-3.28, -1.68] | <0.00001 | 71 | <0.0001 | Robust |
| [24]. Yu et al., 2022 | -2.54 [-3.41, -1.67] | <0.00001 | 71 | <0.0001 | Robust |
| [25]#1. Ryu et al., 2018 | -2.51 [-3.30, -1.71] | <0.00001 | 71 | <0.0001 | Robust |
| [25]#2. Ryu et al., 2018 | -2.45 [-3.25, -1.65] | <0.00001 | 71 | <0.0001 | Robust |
| [25]#3. Ryu et al., 2018 | -2.34 [-3.11, -1.57] | <0.00001 | 70 | <0.0001 | Robust |
| [27]. Kim et al., 2016 | -2.49 [-3.32, -1.67] | <0.00001 | 71 | <0.0001 | Robust |
| [30]. Li et al., 2017 | -2.28 [-3.02, -1.55] | <0.00001 | 68 | <0.0001 | Robust |
| [32]. Xie et al., 2017 | -2.56 [-3.38, -1.74] | <0.00001 | 69 | <0.0001 | Robust |
| [33]. Chung et al., 2019 | -2.52 [-3.34, -1.71] | <0.00001 | 71 | <0.0001 | Robust |
| [34]#1. Lee et al., 2018 | -2.49 [-3.30, -1.69] | <0.00001 | 71 | <0.0001 | Robust |
| [34]#2. Lee et al., 2018 | -2.47 [-3.27, -1.67] | <0.00001 | 71 | <0.0001 | Robust |
| [34]#3. Lee et al., 2018 | -2.40 [-3.20, -1.60] | <0.00001 | 71 | <0.0001 | Robust |

**Table S2b Sensitivity analyses of Fibrosis**

| Omitted study | SMD (95% CI) | ***p***-value  for Overall Effect | Heterogeneity  ***I²*** (%) | Heterogeneity  ***p***-value | Impact  Assessment |
| --- | --- | --- | --- | --- | --- |
| None | -2.13 [-2.67, -1.59] | <0.00001 | 7 | 0.38 | Baseline |
| [17]. Furuta et al., 2018 | -2.04 [-2.56, -1.52] | <0.00001 | 0 | 0.49 | Robust |
| [18]. Hendawy et al., 2022 | -2.06 [-2.59, -1.54] | <0.00001 | 1 | 0.42 | Robust |
| [21]#1. Kim et al., 2017 | -2.23 [-2.76, -1.71] | <0.00001 | 0 | 0.45 | Robust |
| [21]#2. Kim et al., 2017 | -2.23 [-2.79, -1.67] | <0.00001 | 6 | 0.39 | Robust |
| [24]. Yu et al., 2022 | -2.13 [-2.83, -1.42] | <0.00001 | 18 | 0.29 | Robust |
| [27]. Kim et al., 2016 | -2.14 [-2.78, -1.50] | <0.00001 | 18 | 0.28 | Robust |
| [34]#1. Lee et al., 2018 | -2.23 [-2.78, -1.69] | <0.00001 | 3 | 0.41 | Robust |
| [34]#2. Lee et al., 2018 | -2.13 [-2.74, -1.52] | <0.00001 | 18 | 0.29 | Robust |
| [34]#3. Lee et al., 2018 | -2.04 [-2.60, -1.49] | <0.00001 | 5 | 0.39 | Robust |

**Table S2c Sensitivity analyses of Apoptosis**

| Omitted study | SMD (95% CI) | ***p***-value  for Overall Effect | Heterogeneity  ***I²*** (%) | Heterogeneity  ***p***-value | Impact  Assessment |
| --- | --- | --- | --- | --- | --- |
| None | -3.09 [-3.94, -2.23] | <0.00001 | 40 | 0.08 | Baseline |
| [21]#1. Kim et al., 2017 | -3.13 [-4.06, -2.20] | <0.00001 | 46 | 0.05 | Robust |
| [21]#2. Kim et al., 2017 | -3.13 [-4.07, -2.19] | <0.00001 | 46 | 0.05 | Robust |
| [24]. Yu et al., 2022 | -3.10 [-4.14, -2.07] | <0.00001 | 44 | 0.07 | Robust |
| [25]#1. Ryu et al., 2018 | -3.26 [-4.16, -2.36] | <0.00001 | 40 | 0.09 | Robust |
| [25]#2. Ryu et al., 2018 | -3.24 [-4.17, -2.30] | <0.00001 | 43 | 0.07 | Robust |
| [25]#3. Ryu et al., 2018 | -2.95 [-3.81, -2.09] | <0.00001 | 39 | 0.10 | Robust |
| [27]. Kim et al., 2016 | -3.31 [-4.21, -2.40] | <0.00001 | 33 | 0.14 | Robust |
| **[30]. Li et al., 2017** | **-2.89 [-3.60, -2.18]** | **<0.00001** | **19** | **0.27** | **Significantly**  **reduced**  **heterogeneity** |
| [34]#1. Lee et al., 2018 | -3.16 [-4.11, -2.22] | <0.00001 | 46 | 0.05 | Robust |
| [34]#2. Lee et al., 2018 | -3.04 [-3.95, -2.14] | <0.00001 | 45 | 0.06 | Robust |
| [34]#3. Lee et al., 2018 | -2.88 [-3.72, -2.05] | <0.00001 | 34 | 0.14 | Robust |

Notes: Robust indicates that omitting the study did not substantially change the pooled effect estimate or its statistical significance. Significantly reduced heterogeneity indicates that omitting the specific study identified it as a primary source of statistical heterogeneity.
